# Engineering a CEACAM1 Variant with the Increased Binding Affinity to TIM-3 Receptor

**DOI:** 10.61186/ibj.3874

**Published:** 2023-05-28

**Authors:** Zahra Hajihassan, Mehran Mohammadpour Saray, Aysan Yaseri

**Affiliations:** Faculty of New Sciences and Technologies, University of Tehran, Tehran, Iran

**Keywords:** Binding affinity, Mutagenesis, TIM-3 protein

## Abstract

**Background:**

TIM-3 is an inhibitory receptor expressed in a variety of cells, including dendritic cells, T-helper 1 lymphocytes, and natural killer cells. Binding of this protein to its ligand, CEACAM1, causes T-cell exhaustion, a specific condition in which effector T cells lose their ability to proliferate and produce cytokines. Blocking this inhibitory receptor is known to be an effective strategy for treating cancer and other related diseases. Therefore, in this study, in order to block the inhibitory receptor of TIM-3, we designed and produced recombinantly a protein with a high binding affinity to this receptor.

**Methods:**

The extracellular domain of CEACAM1 involved in binding to TIM-3 was mutated using R script to obtain a variant with the increased binding affinity to TIM-3. The binding energy of the mutant protein was calculated using the FoldX module. Finally, after recombinant production of the most appropriate CEACAM1 variant (variant 39) in *E. coli*, its secondary structure was determined by CD spectroscopy.

**Results:**

The binding free energy between variant 39 and TIM-3 decreased from -5.63 to -14.49 kcal/mol, indicating an increased binding affinity to the receptor. Analysis of the secondary structure of this variant also showed that the mutation did not significantly alter the structure of the protein.

**Conclusion:**

Our findings suggest that variant 39 could bind to TIM-3 with a higher binding affinity than the wild-type, making it a proper therapeutic candidate for blocking TIM-3.

## INTRODUCTION

T-cell exhaustion is a specific condition in which effector T cells lose their ability to proliferate and produce cytokines due to the permanent presence of antigen or inflammation in the chronic infectious, autoimmune and cancer diseases^[1]^. One of the critical features of the exhausted T cells is the high-level expression of inhibitory receptors, including programmed cell death-1 (PD-1), cytotoxic T lymphocyte antigen-4 (CTLA-4), lymphocyte-activation gene 3 (LAG3), T cell immunoglobulin and mucin domain-containing protein 3 (TIM-3), V-domain Ig suppressor of T cell activation (VISTA), and T-cell immunoreceptor with Ig and ITIM domain (TIGIT)^[^^2^^]^. The TIM family has three members (TIM-1, TIM-3, and TIM-4) in humans and eight (TIM-1 to TIM-8) in mice^[^^3^^]^. Human TIM-3, also known as HAVCR2 or hepatitis A virus cellular receptor 2, contains 302 amino acids, which include an IgV-like domain involving in ligand binding, a mucin domain, and also a transmembrane domain that links to an intracellular cytoplasmic tail involving in the phosphotyrosine-dependent signaling. This receptor belongs to the immunoglobulin superfamily (IgSF) and plays a critical role in the regulation of innate and adaptive immunity. The protein TIM-3 is expressed in various cells, such as T-helper 1 lymphocytes, dendritic cells, natural killer cells, and macrophages and has four ligands, including phosphatidylserine, CEACAM1, high mobility group box 1 (HMGB1), and galectin-9^[^^4^^,^^5^^]^.

CEACAMs are expressed in leukocytes, as well as in epithelial and endothelial cells, and contribute to various natural and pathological processes, including cancer, inflammation as well as bacterial and viral infections. These proteins have also been reported to be involved in morphogenesis, apoptosis, angiogenesis, cell proliferation, cell motility, fibrosis, and immune tolerance^[^^6^^]^. CEACAM1, a single-pass transmembrane protein type 1, has 12 isoforms. All the isoforms have an IgV domain, which have an essential role in binding to itself and also to other receptors or microorganisms, as well as three constant immunoglobulin type 2 or IgC2 immunoglobulin codomains (A1, B, and A2), a transmembrane sequence and a cytoplasmic signaling domain^[^^7^^]^.

TIM-3 and CEACAM1 form a heterodimeric complex. Biochemical and biophysical studies have shown that the IgV domain has a critical participation in the binding of these two proteins. In chronic viral infections and in the tumor microenvironment, the binding of TIM-3 and CEACAM1 leads to T-cell exhaustion^[^^8^^,^^9^^]^. However, reversing this condition by blocking these inhibitory receptors employing antibodies or peptides is known to be an effective strategy for cancer treatment^[^^8^^]^. In many cases, the use of peptides is superior to antibodies due to their small size, low production cost, and low immunogenicity^[^^10^^]^. Therefore, to block the TIM-3 receptor, we, in this study, mutated the extracellular domain of CEACAM1, which is involved in protein binding, to obtain a variant with high binding affinity to TIM-3.

## MATERIALS AND METHODS

All reagents were purchased from Merck Company (Germany) unless otherwise specified.

**Bioinformatics analysis**

In this study, the sequence of IgV domains of CEACAM1 and TIM-3 with 5DZL and 5F71cods was obtained from the UniProt database (https://www. uniprot.org/), respectively. The complex of TIM-3 and CEACAM1 was constructed using HADDOCK web server (https://wenmr.science.uu.nl/ haddock2.4). Also, the alanine scanning technique and ROBETTA server (http://robetta.bakerlab.org) were used to detect the most important amino acids involving in binding TIM-3 to CEACAM1. Subsequently, mutations were generated at two positions of the CEACAM1 IgV domain using R software (version 3.4.2); the amino acids in these two positions were replaced by 10 amino acids capable of forming hydrogen or ionic bonds. Each protein sequence was then modelled by homology modelling using the SWISS-MODEL server (https://swissmodel. expasy.org/) and minimized using Chimera software (version 1.12). The HADDOCK server was used for the computational docking of the mutant samples with the TIM-3 protein. Finally, FoldX software (YASARA version 17.8.15) was employed to calculate the binding free energy between the protein mutants and TIM-3. The most suitable variant was selected according to the lowest binding energy. In order to analyze and compare the binding sites and interactions between CEACAM1 and TIM-3 in the wild type and mutant complexes, the PDBsum database (https://www.ebi.ac.uk/thornton-srv/software/ PDBsum1) was applied. The MolProbity server, http://molprobity.biochem.duke.edu( was also used to generate the Ramachandran plots.

**Experimental analysis**

***Bacterial strains and culture conditions***

The *SHuffle* *T7* and *Rosetta gami* (DE3) strains of *E. coli* were purchased from Novagen (USA) and used as the host for protein expression. The *SHuffle* *T7* was grown in a Luria–Bertani medium (Sigma, USA) at 30 °C with the addition of 100 µg/ml of ampicillin and streptomycin antibiotics for the selection of recombinant bacteria. *Rosetta gami* strain was grown in the same culture medium with 100 µg/ml of ampicillin at 37 °C. 

***Construction of the expression cassette***

The codon-optimized complementary DNA of the selected variant was synthesized by ShineGene Company (China) and cloned into the pET21a(+) vector (purchased from Novagen) using *Nde*I and *Xho*I restriction enzymes (Jena Bioscience, Germany). Subsequently, the recombinant vector was transformed to the bacteria by CaCl_2_ and the heat-shock procedure^[11]^.

***Protein expression and extraction***

For protein expression, 1% dilution of the overnight culture was inoculated into a fresh medium and incubated until the absorbance at OD_600_ nm reached 0.6. Then 0.7 mM of IPTG (Sigma) was added to the medium, and the cells were grown for additional 16 and 4 h for the *SHuffle* *T7* and *Rosetta gami* strains, respectively^[^^12^^]^. Finally, the cells were collected by centrifugation at 5000 ×g at 4 °C for 15 min. For protein extraction, 8 M of urea was added to the cell pellets, and the resulting suspension was sonicated in 10 repeating cycles for cell disruption. To obtain soluble proteins, the mixture was centrifuged at 4 °C at 12000 ×g for 30 min^[^^13^^]^.

***SDS-PAGE and immunoblot analysis***

For protein electrophoresis, 12% SDS-PAGE was used^[^^14^^]^. Western blotting was performed as described previously^[^^15^^]^. For this purpose, an anti-His tag monoclonal antibody conjugated with horseradish peroxidase (Sigma) was used at a dilution of 1:2000 in a TBS-T buffer (50 mM of Tris-HCl, 150 mM of NaCl, 5% Tween 20) containing 3% w/v skimmed milk. Finally, protein detection was performed using a solution of DAB (Biobasic, Canada) and hydrogen peroxide as enzyme substrates.

***Protein purification***

Purification of the recombinant protein with a poly-histidine tag at the C-terminal was performed using Ni^+2^-NTA affinity chromatography (Agarose Bead Technologies, Spain)^[^^16^^]^.

***Secondary structure determination***

Concentration of the purified protein was determined by the Bradford assay^[^^17^^]^. The far-UV CD spectrum of the purified protein dissolved in 10 mM Tris-HCl (pH 7.4) was recorded using a CD spectrophotometer model 215 (Aviv Instruments Inc., USA). The scan speed was 20 nm/min, and the response time was 0.33030 s with a bandwidth of 1 nm. Quartz cell with a path length of 10 mm was used, and all measurements were carried out at room temperature.

## RESULTS AND DISCUSSION

Immunotherapy is one of the newest strategies used to treat various types of cancer. The use of monoclonal antibodies to block inhibitory receptors on the surface of T cells and other immune cells is commonly used in immunotherapy^[^^18^^]^. However, employment of monoclonal antibodies is not only cost-benefit but also has temporary side effects such as pain, fever, and vomiting, and even more serious adverse events such as neutropenia, hypersensitivity reactions, and upper respiratory tract infections, leading scientists to turn their attention to the production of the small peptide or protein molecules instead of large antibodies^[^^19^^]^. In 2015, Maute and colleagues^[^^20^^] ^designed small peptides, instead of using antibodies, to block PD-L1, an inhibitory ligand for the PD-1 receptor found on the activated T cells. These small peptides are able to block inhibitory responses and have high permeability into tumors. In this study, we designed a variant of CEACAM1 with high binding affinity for TIM-3 to block another inhibitory receptor on the surface of T cells called TIM-3.

**Bioinformatics analysis and mutagenesis**

**Fig. 1:**
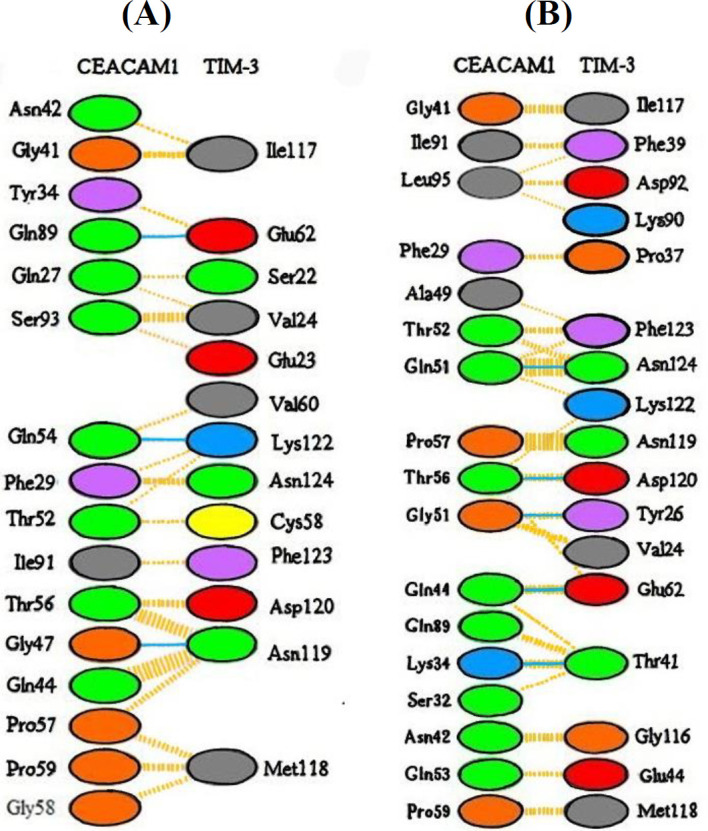
Binding sites of CEACAM1-TIM-3 complex determined by PDBsum server. (A) and (B) are wild-type complex and complex 39 (mutant one), respectively. The orange bonds represent the van der Waals interactions; the greater the number of atoms involved in this interaction, the larger the diameter of these orange bonds. Blue bonds indicate hydrogen forces. Proline and glycine are shown in orange in this diagram. Aromatic and aliphatic amino acids are also displayed in purple and gray, respectively. The amino acids shown in blue and red are amino acids with positive and negative charges, respectively, and the amino acids shown in light green are neutral. Cysteine residue is shown in yellow.

**Fig. 2:**
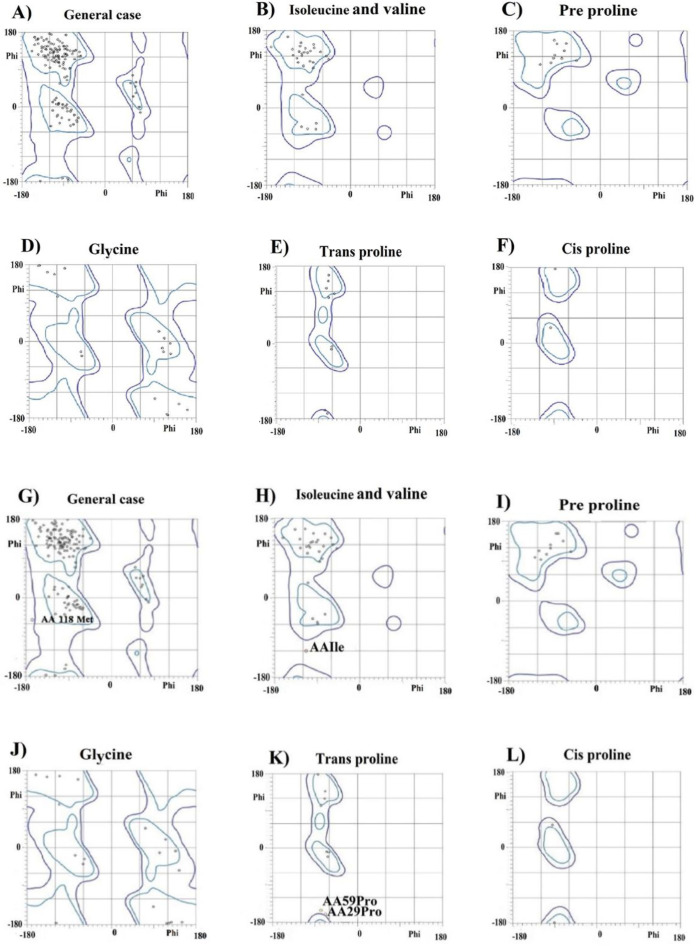
Ramachandran plots for CEACAM1-TIM-3 complex. Chain A and B are TIM-3 and CEACAM1, respectively; (A-F) show the Ramachandran plots of complex 39 with 94.3% amino acids in the favored regions, and (G-L) show Ramachandran plots for wild-type complex of CEACAM1-TIM-3, in which 90.9% of amino acids are in the favored regions.

Evidence has revealed that only the extracellular domain of CEACAM1 is sufficient for its binding to TIM-3^[^^9^^]^; therefore, we selected the sequence of this domain for protein engineering. For this purpose, three-dimensional structures and binding sites of TIM-3 and CEACAM1 proteins were obtained from PDB and UniProt databases, and then alanine scanning was performed to identify the most appropriate protein hotspots. According to the alanine scanning results, by replacing alanine with amino acids at positions 29, 95, 56, 27, 54, 34, and 89, the changes of binding free energy changes were very positive (Table 1). This observation indicates the great importance of these amino acids in the interaction of the two proteins. Among these binding sites, positions 56 and 34, both have positive ΔΔG and have been reported as important binding sites in previous research^[^^6^^]^. It is worth noting that according to the former results, mutations in these regions may cause the loss of structure and function of the entire protein. However, it has been shown that in some cases, these mutations not only have no lethal consequences but also can be effective in creating new variants of proteins with higher binding efficiency and better performance^[^^21^^]^. Thus, using R script, the extracellular domain (IgV) of CEACAM1 was mutated at positions 34 and 56 to increase its binding affinity for the receptor (TIM-3). To this end, amino acids with the ability to form hydrogen or ionic bonds like serine, threonine, tyrosine, glutamine, asparagine, arginine, lysine, histidine, aspartate, and glutamate were selected for substitution in these two positions. Finally, the binding free energy between TIM-3 and each mutant protein was calculated using FoldX software. Four complexes had the lowest binding energy compared to the wild-type complex. Among these four complexes, the one with the lowest binding energy, complex 39, was selected for further experiments. In this complex, amino acid 34 (tyrosine) was replaced by lysine, and amino acid 56 remained unchanged (Table 2). In this mutant (complex 39) with the Y34K point mutation, the binding energy of the protein decreased from -5.63 kcal/mol to -14.49 kcal/mol. Notably, tyrosine 34, one of the key amino acids involving in binding in complex 39, was mutated and replaced by lysine, which is structurally similar to tyrosine. The most important interactions between native CEACAM1 and TIM-3, as well as in the complex39, were evaluated using the PDBsum server. As illustrated in Figure 1, the interaction of these proteins consists mainly of van der Waals and hydrogen bonds. According to the results, the number of hydrogen bonds between the two proteins was 3 in the wild-type complex and increased to 5 after mutagenesis in complex 39. In addition, the total number of van der Waals interactions increased in the mutant complex. The increased number of bonds, especially hydrogen bondsas strong bonds, is the main reason for the greater stability of complex 39 than the wild-type complex. Ramachandran plots (Fig. 2) also confirmed the stability of the mutant complex compared to the wild type, as there were no outlier amino acids after mutagenesis.

**Table 1:** Effects of alanine replacement on CEACAM1 protein binding sites obtained by alanine scanning technique

Hot spots	ΔΔG (kcal/mol)
**56**	0.94
**34**	0.75
**89**	0.66
**27**	0.85
**29**	2.03
**40**	-0.11
**44**	0.30
**52**	0.33
**54**	0.82
**91**	0.44
**95**	1.46

**Table 2:** Binding energy of different complexes versus wild-type complex

**Complex**	**Wild type**	**39**	**95**	**26**	**18**
Binding energy (kcal/mol)	-5.63	-14.49	-12.47	-12.37	-12
Position 34	Tyrosine	Lysine	Tyrosine	Histidine	Glutamic acid
Position 56	Threonine	Threonine	Asparagine	Glutamine	Serine

**Protein expression and purification**

**Fig. 3:**
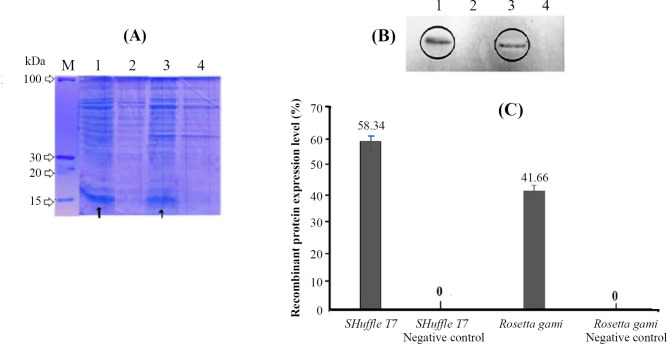
Protein analysis using (A) 12% SDS gel electrophoresis and (B) Western blotting. M, the molecular weight marker; lane 1, proteins extracted from *SHuffle T7*; lane 2, negative control (proteins extracted from *SHuffle T7* without recombinant vector); lane 3, proteins extracted from *Rosetta* *gami*; lane 4, negative control (proteins extracted from *Rosetta* *gami *without recombinant vector)*. *Comparison of protein expression level in* SHuffle T7 *and* Rosetta* *gami *strains calculated by ImageJ software (C). The arrows indicate the protein band corresponding to mutant 39.

**Fig. 4:**
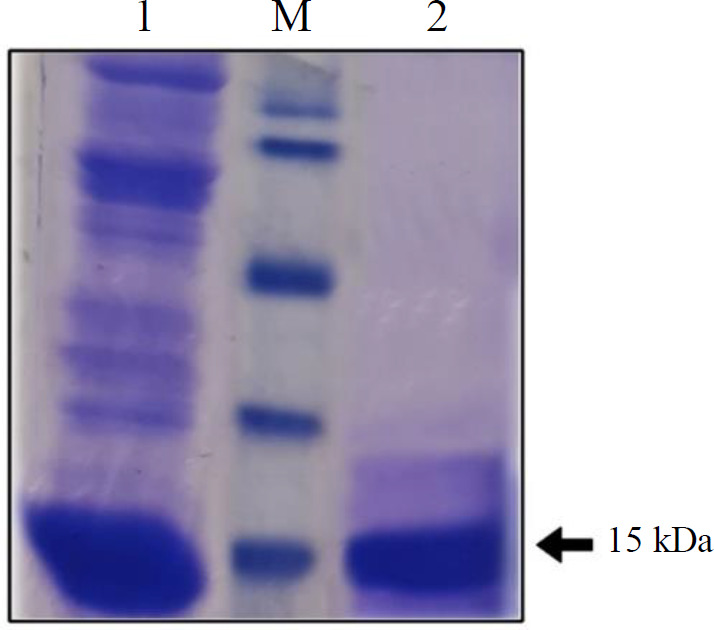
Protein analysis using 12% SDS gel electrophoresis. M, the molecular weight marker; lanes 1 and 2, proteins extracted from *SHuffle T7 *strain before and after purification by Ni^+2^-NTA chromatography, respectively.

Mutant 39 was considered as the most appropriate variant of CEACAM1; therefore, its codon-optimized complementary DNA was synthesized and cloned into pET21a(+) vector and subsequently transformed into two strains of *E. coli*, including *SHuffle* *T7* and Rosetta gami*,* for protein expression. Since the extracellular domain of CEACAM1 has three disulfide bonds, the above-mentioned strains were selected for protein expression because they are two engineered *E. coli *strains that are suitable for soluble expression of the proteins with disulfide bonds in the cytoplasm of *E. coli*. The cytoplasm of *E. coli *is constantly maintained as a reducing environment; hence, it is not suitable for the formation of disulfide bonds. However, due to mutations in the glutaredoxin reductase and thioredoxin reductase enzymes in *SHuffle* *T7* and Rosetta gami *strains*, disulfide bond formation is possible in the cytoplasm of these strains. In addition, *SHuffle* *T7* constitutively expresses a chromosomal copy of the disulfide bond isomerase DsbC, which facilitates disulfide bond formation. The Rosetta gami host strain also allows for the enhanced expression of eukaryotic proteins containing codons rarely used in *E. coli*^[^^22^^]^. After the induction of the promoter using IPTG, SDS- PAGE and Western blotting techniques were applied to confirm the protein expression. The protein band related to the recombinant protein (mutant 39) was located in the 15 kDa region (Fig. 3A). Relevant bands in the Western blotting paper (Fig. 3B) were then examined and quantified using ImageJ software. As represented in Figure 3C, protein was well expressed in both strains. However, the *SHuffle* *T7* strain was slightly higher in terms of expression level and then used for further large-scale production. Finally, the recombinant protein produced in this strain was successfully purified by Ni^+2^-NTA chromatography (Fig. 4).

**Fig. 5:**
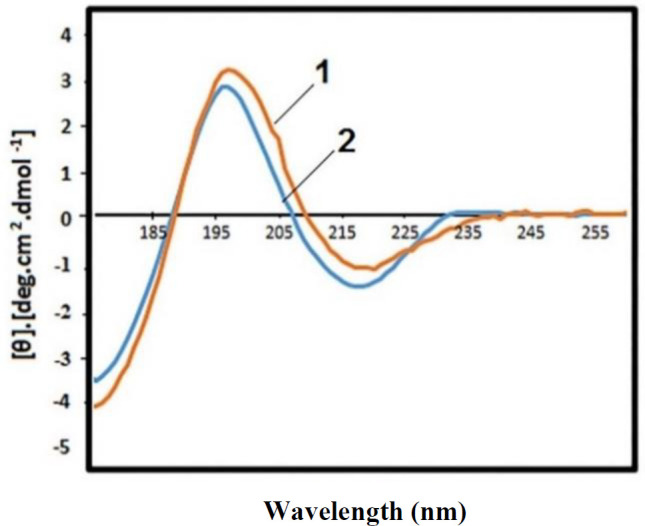
Far-UV CD spectra of wild type CEACAM1 (1) and variant 39 (2). Each spectrum obtained at room temperature with a 10 mm path length cell.

**Table 3:** Comparison of the secondary structure elements of variant 39 and native CEACAM1 reported in the Uniprot

Secondary structure	Mutant 39	Wild type
**Helix**	3.4	0.7
**Anti-Parallel**	47.2	48.2
**parallel**	0.0	0.0
**Turn**	8.6	11.3
**Others**	40	40

**Structural analysis**

In order to determine the secondary structure of the mutant CEACAM1 and also study the effect of the Y34K point mutation in this variant, the far-UV CD spectrum of this mutant protein was obtained by  CD  spectroscopy. Furthermore, this structure was compared with the secondary structure of the extracellular domain of native CEACAM1 reported in the UniProt (Fig. 5). As depicted in Table 3, the protein maintained its structure after the mutation. The highest effect of the mutation was observed on the α-helix structure, i.e. the helix percentage from 0.7 to 3.4 increased. Importantly, given the relationship between protein structure and function^[^^23^^]^, the mutant protein with the correct secondary structure is expected to retain its function and could be a suitable candidate for immunotherapy studies. 

## Conclusion

In this study, we described a variant of the CEACAM1 protein with the increased binding affinity to the TIM-3 receptor. Among 100 variants in our library, this variant was the most suitable in terms of binding to TIM3. Using recombinant DNA technology, this mutant protein was expressed in *E. coli*, and finallyits secondary structure was determined by CD spectroscopy. Since its structure was reasonably similar to that found in UniProt for native CEACAM1, it could be considered as a candidate for future immunotherapy studies. Binding of this variant to TIM3 likely blocks the inhibitory response that can be transmitted from TIM3 to T cells. 

## DECLARATIONS

### Acknowledgments

The authors would like to acknowledge the financial support of University of Tehran under Grant Number 28669/06/15.

### Ethical statement

Not applicable.

### Data availability

The raw data supporting the conclusions of this article are available from the authors upon reasonable request. 

### Author contributions

ZH: conceptualization, analyzing data, and writing final draft; MMS: investigation and writing first draft; AY: investigation and writing first draft

### Conflict of interest

None declared.

### Funding/support

This work was supported by the University of Tehran, Tehran, Iran under Grant Number 28669/06/15.
